# On Regimen and Longevity; Comprising Materia Alimentaria, National Dietetic Usages, and the Influence of Civilization on Health and the Duration of Life

**Published:** 1844-07

**Authors:** 


### Art. VIII.

*On Regimen and Longevity; comprising Materia Alimentaria, National Dietetic Usages, and the Influence of Civilization on Health and the Duration of Life.* By John Bell, m.d. — *Philadelphia,* 1842. 12mo, pp. 420.

We should have included this work in our leading article of No. XXXIII, had it reached us in time. The author addresses himself chiefly to the general reader, calculating also upon the advantages, as we learn from the preface, that the physician may obtain from his treatise. There can be little doubt of the propriety of instructing the multitude on the subject of food and diet, as a branch of hygiene; and the literary productions intended to fulfil this purpose come properly under our consideration. For several very obvious reasons, they demand the most jealous scrutiny. To the practitioner of the present day, if one thing be of more real importance, than another it is that the prejudices of society on the general subject of regimen should be dispelled by education, and the judicious writings of physicians are best calculated to effect this object. But the author who thus writes for the public can rarely, at the same time, expect to enlighten his brethren. In the capacity of a popular author he may well express his hope, that the matter laid before the reader may meet with the approval of the profession, and he ought to be extremely careful to secure that object. If, on the other hand, a work be intended to advance medical knowledge, by propounding new principles, or by novel applications of those that are already established, or if it encroach upon the proper province of therapeutics, it should relinquish entirely its popular character. In this case it is liable to become not merely useless, but most mischievous in the hands of the non-medical reader.

Dr. Bell's is a strictly popular work. At the same time it comprises many most interesting facts derived from various works on the physical history of mankind, on political economy and statistics, and from voyages and travels, the knowledge of which is not sufficiently diffused among medical practitioners. The author extols the advantages of agriculture to a country, and the influence of abundance of food over its inhabitants:

"While advocating simplicity, he also recommends variety in dietetic regimen: he thinks that meat should be sparingly used, but he displays the endless variety of vegetable food, and the prodigal supply of fruits, with a luxurious enjoyment in their free use,—in the state in which, by a favouring climate, and skilful industry, they are met with in nearly all parts of the world. ...... Horticulture, the attendant and embellisher of agriculture, which provides so many palatable and healthful additions to the substantial produce of the field, and correctors of the undue stimulus and acrimony of much animal food, merits all the fostering care which an uncorrupted and yet educated and refined taste has ever extended to it. A well-cultivated garden, in due alternations of vegetable, fruit, and flower, gives us poetry without its allusions, nature divested of her ruggedness, and art of its constraint." (p. vii.)

Dr. Bell is strongly in favour of a diet consisting mainly of vegetables. The chapters on National Dietetic Usages, although little more than a collection of facts from the sources above mentioned, with but little reasoning or deduction, and none of that which the readers of our review of Dr. Pereira's work will understand as the science of the subject, are still worthy of perusal. In favour of a vegetable diet the Athenians and the ancient Greeks generally, the ancient and modern Egyptians, the wandering Arabs, and the Persians, and the peasantry for the most part of modern Europe are quoted. The Hindoo is also contrasted with the Esquimaux, the Chinese with the Laplanders, and the agricultural inhabitants of Mexico and the South with the carnivorous North American Indians. The author enters rather fully into the subject of fruits. He illustrates and dwells much upon the fact of an abundance of vegetable food, more particularly in favouring an increase of population. He regards the employment of corn and potatoes in distilling, as the "destruction of human food." He laments the excesses of the Americans in eating, and condemns, in no measured terms, their habitual drunkenness. He inculcates temperance to the letter, and is an enemy to the use of alcoholic fluids of every description.

We hope, at no very distant period, to bring before our readers the whole question respecting the use of alcoholic fluids, and shall accordingly forbear alluding more particularly to the author's views on the present occason, than by giving the estimate he has made of "the real state of French temperance,"and we may add of American intemperance:

"The sum total of alcohol in the different drinks used by the French people is as follows:

| *Gallons.* | | | *Gallons of Alcohol.* |
|---|---|---|---|
| In 611,466,000 | of wine, | at 9½ per cent. | 55,000,000 |
| 234,121,000 | of cider, | at 7 ,, | 16,388,470 |
| 124,000,000 | of beer, | at 5 ,, | 6,200,000 |
| 17,954,800 | of brandy, | at 50 ,, | 8,982,400 |
| 987,551,800, | averaging 8·7 per cent. | | 86,570,870 |

This gives more than two gallons and a half per annum of pure alcohol for every individual in France. After making certain deductions, 7·7 gallons annually per man. By a similar calculation, the consumption per individual for Great Britain and Ireland is 1·28 gallons annually; and for the United States 5½ gallons, without taking into the account the wine, beer, and cider consumed in the latter country. These estimates are drawn from statistical documents of the year 1830. From similar documents of 1840 it would appear that the whole of the alcohol consumed in America amounted to 3¼ gallons annually for every individual in the States; being a reduction in the period of ten years of about 57 per cent.

Two hundred and fifty-five pages of the volume are occupied in the descriptions of the various articles employed as food. The period of publication precluded the author's acquaintance with the principles of dietetics promulgated of late on the continent and in this country. Our readers, therefore, will not expect us to open anew a subject that has been recently so fully treated of in this Journal, and we may content ourselves by stating, that, having examined the facts contained in the work before us, we find them upon the whole illustrative and confirmatory of the principles set forth in our review of Dr. Pereira's treatise.

On the subject of Longevity, we find Quetelet and Lombard quoted, and the author is indebted for his materials generally to Villermé, Edwards, Southwood Smith, Humboldt, Bulwer, Farr, the Reports on the Poor Laws, and of the Committee of Inquiry into Drunkenness, &c. &c., which authors and works are severally quoted. Dr. M'Culloch's 'Commercial Dictionary' has also been laid under heavy contribution.

The specimen we have given of the author's style is a favorable one. Throughout the work his sentences are frequently obscure and faulty in their construction. We meet with new words of American coinage, as "outranks," "evincive," &c. Others are misapplied, as where it is stated that magnesia is more "salubrious" than potash.

On the whole, however, we are bound to state that, as a popular work, advocating moderation and temperance, and, so far as they go, principles of dietetics in the main correct, Dr. Bell's work is well calculated to diffuse information and to do good to his fellow-men both in America and Europe.

